# Supplementary material for: TEM Investigation of Asymmetric Deposition-Driven Crystalline-to-Amorphous Transition in Silicon Nanowires
Source: Materials (Basel). 2022 Oct 12;15(20):7077. doi: 10.3390/ma15207077 (PMC9604709; doi:10.3390/ma15207077)
Supplement: Supplementary file 1 [file materials-15-07077-s001.zip › materials-1906127-supplementary.pdf]

Produced by L.B. Li et al.

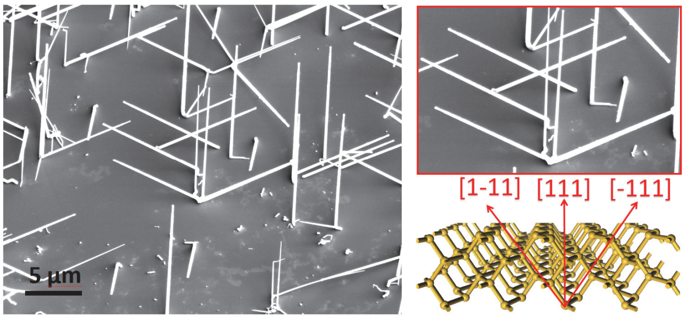

Figure S1 SEM images of SiNWs grown with Au colloid and the schematic of growth orientations of the SiNWs. SiNWs were prepared on n-type (doping concentration of 10^{17} cm^{-3}) on-axis Si(111) substrate by LPCVD. The substrates were cleaned with acetone and IPA before polymer and colloid deposition. $SiCl_4$ was used as the precursor molecule for SiNWs growth. H_2 (15%) in argon was used as the carrier gas flowed through the Si precursor bath and into the reaction tube. Au colloids with a diameter of 100~250nm were used to define the diameter and position of the SiNWs.

Produced by L.B. Li et al.

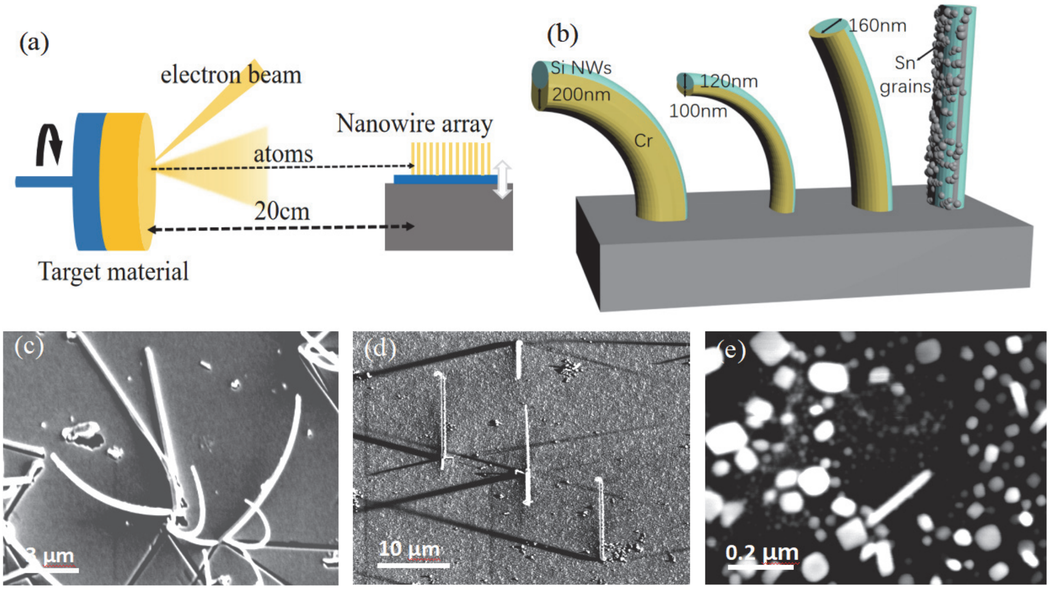

Figure S2 Bent Si NWs fabricated by using sideways electron beam evaporation. Schematic of the experimental set up (a) and SiNWs with asymmetric Cr and Sn (b), SEM images of Si NWs with 100 nm asymmetric Cr layer (c) and Sn layer (d-e). With the asymmetric growth of Cr, Si NWs were bent toward the direction which the deposition was made and became arc-shaped nanostructures.

Produced by L.B. Li et al.

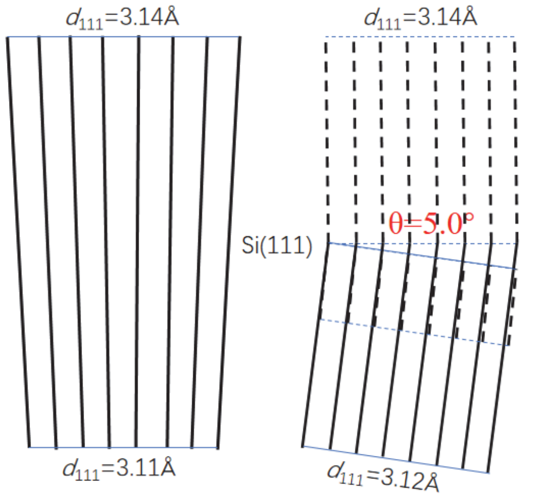

Figure S3 Tensile strain release by orientation rotation in the Si(111) crystalline planes.

Produced by L.B. Li et al.

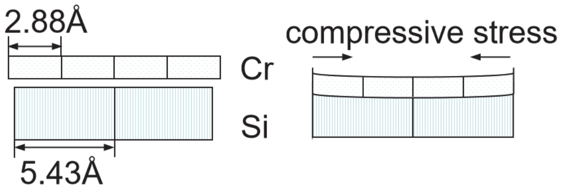

Figure S4 Schematic of lattice stress induced bending of Si NWs with asymmetric Cr.

Produced by L.B. Li et al.

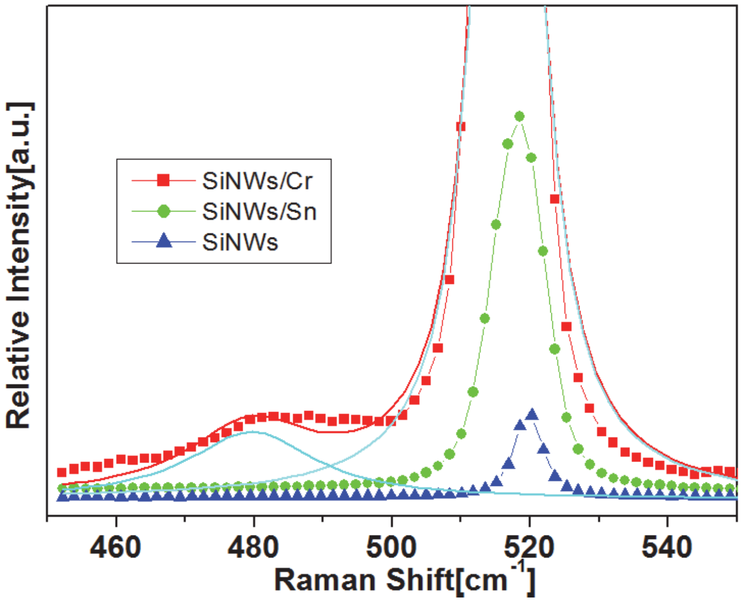

Figure S5 Raman spectra of Si NWs with different asymmetrical layers.

Produced by L.B. Li et al.

Table S1 Coefficient of thermal expansion and lattice constant of Si, Cr and Sn

	Si	Cr	Sn
Coefficient of Thermal Expansion	2.5×10^{-6}/K	6.2×10^{-6}/K	2.0×10^{-6}/K
Lattice Constant	5.43Å	2.88Å	6.49Å
Crystal Structure	FCC	BCC	BCC
